# Targeted *In Vivo* Extracellular Matrix Formation Promotes Neovascularization in a Rodent Model of Myocardial Infarction

**DOI:** 10.1371/journal.pone.0010384

**Published:** 2010-04-28

**Authors:** Shirley S. Mihardja, Dongwei Gao, Richard E. Sievers, Qizhi Fang, Jinjin Feng, Jianming Wang, Henry F. Vanbrocklin, James W. Larrick, Manley Huang, Michael Dae, Randall J. Lee

**Affiliations:** 1 Cardiovascular Research Institute, University of California San Francisco, San Francisco, California, United States of America; 2 Department of Medicine, University of California San Francisco, San Francisco, California, United States of America; 3 Institute for Regeneration Medicine, University of California San Francisco, San Francisco, California, United States of America; 4 Department of Radiology, University of California San Francisco, San Francisco, California, United States of America; 5 UCSF/UCB Joint Bioengineering Graduate Group, University of California, Berkeley, California, United States of America; 6 UCSF/UCB Joint Bioengineering Graduate Group, University of California San Francisco, San Francisco, California, United States of America; 7 Panorama Research Institute, Sunnyvale, California, United States of America; Tufts University, United States of America

## Abstract

**Background:**

The extracellular matrix plays an important role in tissue regeneration. We investigated whether extracellular matrix protein fragments could be targeted with antibodies to ischemically injured myocardium to promote angiogenesis and myocardial repair.

**Methodology/Principal Findings:**

Four peptides, 2 derived from fibronectin and 2 derived from Type IV Collagen, were assessed for in vitro and in vivo tendencies for angiogenesis. Three of the four peptides—Hep I, Hep III, RGD—were identified and shown to increase endothelial cell attachment, proliferation, migration and cell activation *in vitro*. By chemically conjugating these peptides to an anti-myosin heavy chain antibody, the peptides could be administered intravenously and specifically targeted to the site of the myocardial infarction. When administered into Sprague-Dawley rats that underwent ischemia-reperfusion myocardial infarction, these peptides produced statistically significantly higher levels of angiogenesis and arteriogenesis 6 weeks post treatment.

**Conclusions/Significance:**

We demonstrated that antibody-targeted ECM-derived peptides alone can be used to sufficiently alter the extracellular matrix microenvironment to induce a dramatic angiogenic response in the myocardial infarct area. Our results indicate a potentially new non-invasive strategy for repairing damaged tissue, as well as a novel tool for investigating *in vivo* cell biology.

## Introduction

Recent advances in the field of stem cell therapy have renewed enthusiasm for the prospects of myocardial regeneration and repair. Much research has been dedicated into fully assessing the potential of cell therapy in promoting tissue regeneration. However, certain hurdles need to be resolved in order to optimize cell therapy for myocardial regeneration. One of these challenges involves providing the cells a sufficient environment for proper engraftment, sustainability and induction of differentiation [1], [2].

The extracellular matrix (ECM) plays an important role in cell engraftment and tissue regeneration. The development of biocompatible scaffolds acting as an extracellular matrix to serve as a substrate for sustaining cell growth, survival, differentiation, and other biologically relevant functions has become an integral aspect of tissue engineering. In this study, we hypothesized that an *in vivo* matrix could be formed by targeting ECM fragments to an area of myocardial injury and facilitate myocardial repair. To test this hypothesis, we determined whether the composition of the ECM in the region of a myocardial infarct could be altered to promote neovascularization.

Even in the presence of angiogenic cytokines such as vascular endothelial growth factor (VEGF), endothelial cells (ECs) require adhesion to the ECM to facilitate migration. Migration of ECs plays an important role in angiogenesis via sprouting of new blood vessels from the existing vasculature [3]. The maturation of vessels is dependent on the establishment of a continuous basement membrane [4]. The ECM, which consists of structural proteins (e.g. collagen), adhesive proteins (e.g. fibronectin, FN), anti-adhesive proteins (e.g. tenascin), and proteoglycans [5], plays a pivotal role in the activation of various intracellular signaling pathways that are involved in cell migration, survival, proliferation, differentiation, and angiogenesis [6]. The composition of the ECM is constantly changing in order to direct the growth, migration, and differentiation of the ECs into blood vessels. For instance, in the early stages of angiogenesis, type IV collagen (Col IV) appears in patchy subendothelial deposits, which correlates with lumen formation and maintenance, but in the later stages Col IV appears as a continuous mesh, which may act to prevent vascular regression and promote maintenance of the newly formed vessel [7], [8], [9]. Additionally, it has been suggested that degradation of the basement membrane facilitates exposure to collagen and fibrinogen to encourage sprouting and initiation of capillary morphogenesis with the maturation of the vessel lumen occurring with the re-establishment of the intact basement membrane [3].

Here, we investigated whether functional groups derived from Col IV [10], [11], [12] and FN [13], [14], [15], [16] (Table 1) could sufficiently alter the microenvironment to favor neovascularization. Col IV is a major component of the basement membrane and has been shown to promote and regulate the formation, elongation, and stabilization of microvessels during angiogenesis [7]. FN is a major component of the ECM and is known to be involved in promoting wound healing by recruiting endothelial or epithelial cells to the site of injury [17]. The ECM-derived functional groups were chemically conjugated to a monoclonal antibody targeting an injury-specific antigen within the MI, thereby allowing us to non-invasively deliver the ECM to the site of injury.

**Table 1:** Sequence of the peptides along with their source protein.

Peptide Name	Sequence	Protein of Origin
HepI [10], [12]	TAGSCLRKFSTMY-OH	Collagen IV
HepIII [10], [11]	GEFYFDLRLKGDKY-OH	Collagen IV
FC/HV [13], [16]	WQPPRARI-OH	Fibronectin
RGD [14], [15]	GRGDSPASSPISC-OH	Fibronectin

## Results

### *In vitro* cell attachment, proliferation, and migration

**Figure 1:**
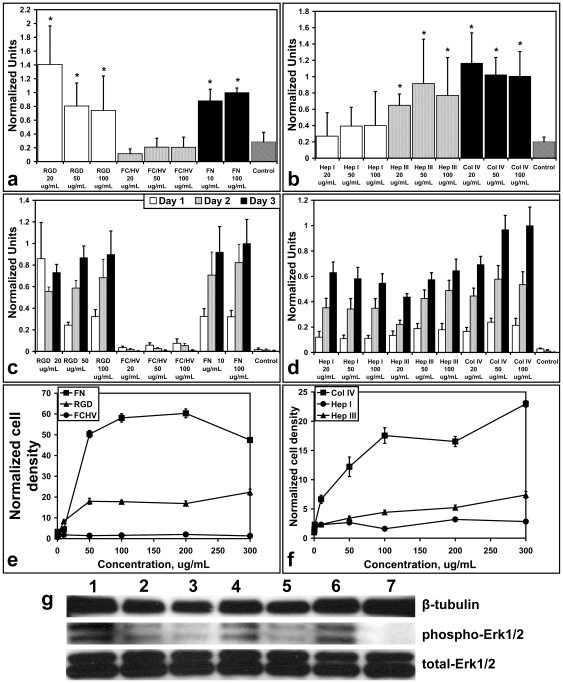
*In vitro* assays. (**a**) and (**b**) Cell adhesion was assessed after 30 minutes of incubation in wells coated with peptides at 20 µg/mL, 50 µg/mL, and 100 µg/mL. The absorbance readings were normalized to either 100 µg/mL FN or 100 µg/mL Col IV, depending on the peptide's source protein, to allow for comparison. * P<0.05. (**c**) and (**d**) Cell proliferation was also assessed with peptides at 20 µg/mL, 50 µg/mL, and 100 µg/mL at Day 1 (white), Day 2 (gray), and Day 3 (black). The absorbance readings again were normalized to either 100 µg/mL FN or 100 µg/mL Col IV, depending on the peptide's source protein. (**e**) and (**f**) Haptotactic migration at various peptide or protein concentrations. The area cell densities have been normalized to allow for comparison. (**g**) Western blot analysis showed phosphorylation of Erk1/2 in cells grown on HepI, HepIII, Col IV, RGD, FC/HV, and FN (Wells 1–6, respectively). No phosphorylated Erk1/2 band was seen for cells grown on PBS-treated dishes (Well 7). Total Erk1/2 was present in the cells grown under all the conditions. β-tubulin was used as an internal control.

To investigate the biological activity of the ECM peptides, the peptides were compared to their full length protein in cell adhesion and cell proliferation assays. The RGD and HepIII peptides showed initial cell adhesion significantly better relative to wells treated with only phosphate buffered saline (PBS) (Figure 1a–b). Cell proliferation (Figure 1c–d) was observed for HepI, HepIII, and RGD, but not for FC/HV.

Gradients of immobilized ECM components have been shown to drive haptotactic migration *in vitro*, which is not dependent on cytokines [18], [19]. Although the importance of this ability has not been fully assessed, it stands to reason that, *in vivo*, higher concentrations of certain ECM components encountered by endothelial cells during new vessel formation may direct their recruitment and outward migration partly via haptotaxis [3]. Hence, we sought to assess the peptides' ability to recruit cells by looking at their ability to induce haptotactic migration (Figures 1e–f). Peptides HepI, HepIII, and RGD promoted haptotactic migration, but to a lesser extent compared to their protein counterparts. Migration was better with HepIII than with HepI. Both HepIII and RGD were able to promote statistically significant migration, as determined by the area cell density, when compared to the membrane coated with only PBS even at concentrations as low as 0.5 µg/mL. For HepI, the migration was statistically significantly higher at concentrations ≥1 µg/mL. No migration was observed with FC/HV.

Cell morphology on the various peptide coatings were compared to their source protein counterparts (Figure S1). Only cells with round morphology were observed on the PBS-treated dishes, indicating that they had not properly adhered to the dish surface. Cell morphology on dishes coated with RGD, HepI, or HepIII was similar to the cell morphology on the Col IV- or FN-coated dishes, i.e. spread out cells, indicating adherence to the dish surface. There were some adherent cells after 24 hours of incubation in the FC/HV-coated dishes, but these adherent cells were no longer observed in subsequent days.

### Activation of Erk1/2 by ECM peptides

All ECM peptides induced, although to varying degrees, activation(Figure 1g) —i.e. phosphorylation—of extracellular signal-regulated kinase 1/2 (Erk1/2), which is involved in the signaling pathway leading to angiogenesis and arteriogenesis [20]. We saw no activation of Erk1/2 for cells cultured on the PBS-treated dishes.

### Antibody targeting of ECM peptides to MI

**Figure 2:**
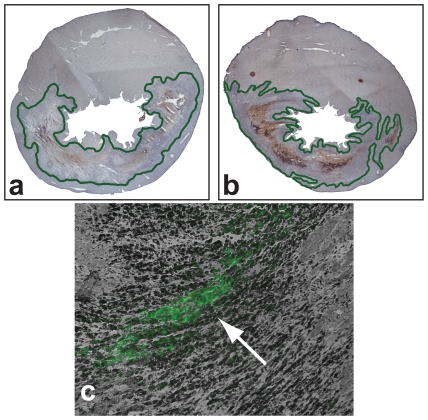
Immunostaining for Ab-HepIII in the infarct region. Hearts from rats sacrificed on 1 day after injection. The rats were injected intravenously with either (**a**) PBS or (**b**) the Ab-HepIII 1 day post-MI. The presence of the Ab is indicated by the brown stain. For the sake of clarity, we have outlined the infarct region. At high magnification, we were also able to see fluorescence within the infarct region(**c**), as indicated by the arrow, of the FITC-labeled peptides (indicated in black).

To test whether we could direct the ECM-derived peptides to the injured myocardium, HepIII was conjugated to anti-rat cardiac α-myosin heavy chain (anti-MHC) using carbodiimide chemistry. Two days post-MI, either HepIII conjugated to the antibody (Ab-HepIII) or PBS (control) were injected via the external jugular vein into Sprague-Dawley rats, who were sacrificed 24 hours later. We stained for the presence of the antibody (Ab) using Mouse-on-rat HRP-polymer (Biocare Medical, Concord, CA). We observed positive staining (dark brown) within the MI for the Ab-HepIII-treated heart (Figure 2b). In addition, we had used FITC-labeled peptides. Under fluorescent microscopy, we were able to confirm that the peptides were also present within the MI (Figure 2c), verifying that conjugating the peptide to the Ab allowed us to successfully target the peptide to the MI.

A series of *in vitro* control studies to assess the functional behavior of the peptides conjugated to the Ab (Ab-peptides) also were performed (data not shown). Ab-RGD and Ab-HepIII promoted significantly more initial cell attachment compared to PBS control. Ab-HepI, Ab-HepIII, and Ab-RGD also demonstrated significantly increased proliferation and migration compared to PBS and Ab only controls. Hence, in the case of initial cell attachment and cell migration, there did not appear to be any significant difference between Ab-HepI, Ab-HepIII, Ab-RGD and their unconjugated counterparts. These results are in agreement with previously published reports, suggesting that conjugation of an ECM peptide to ovalbumin or a polymer matrix did not negatively affect its ability to promote cell attachment, proliferation, and migration [10], [11], [14], [15], [16]. Also, endotoxin analysis of the Ab-peptides showed that their endotoxin levels were <0.06 EU/mL, below the FDA limits of 0.5 EU/mL [21].

### Targeted ECM peptides induce angiogenesis

**Figure 3:**
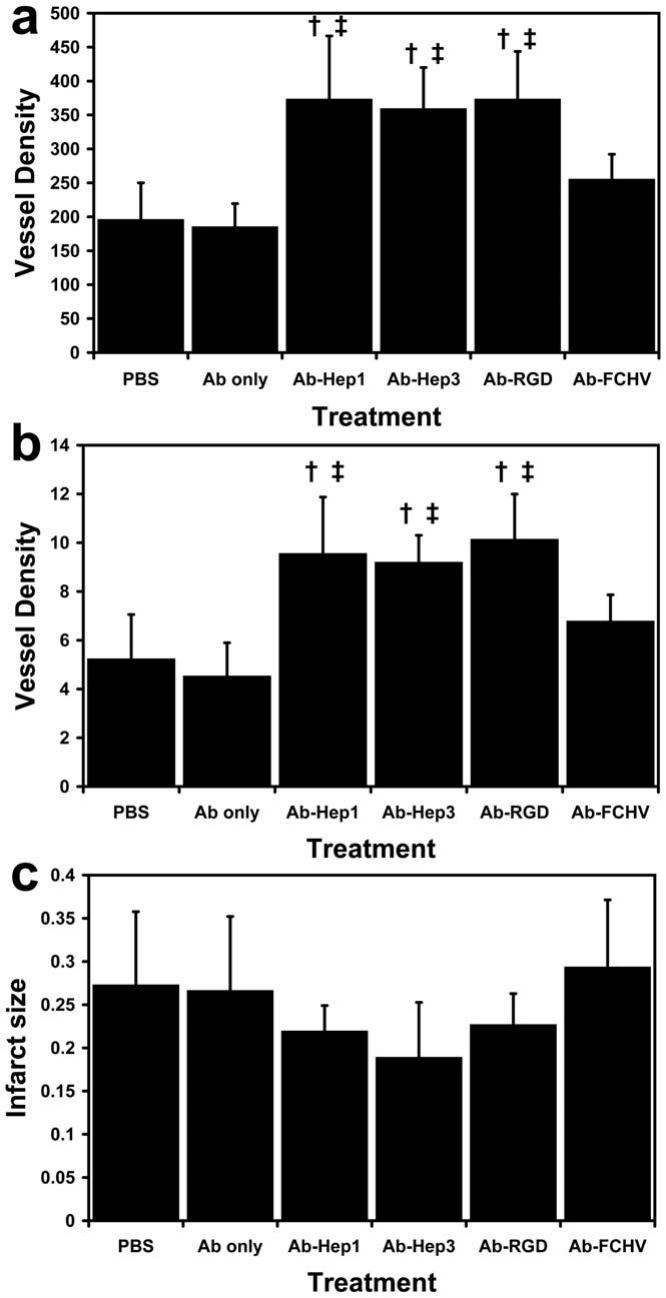
Increased neovascularization within the MI region. (**a**) Capillary staining showed significantly higher capillary density for rats treated with Ab-HepI, Ab-HepIII, or Ab-RGD. † P<0.05 vs. PBS, ‡ P<0.05 vs. Ab-FC/HV. (**b**) Arteriole staining also showed significantly higher arteriole density for rats treated with Ab-HepI, Ab-HepIII, or Ab-RGD. † P<0.05 vs. PBS, ‡ P<0.05 vs. Ab-FC/HV. (**c**) Infarct size measurements showed no statistically significant differences between the treatment groups and the PBS treatment group.

Assessment of capillary formation in the infarct region (Figure 3a), showed that rats treated with Ab-HepI (373±93 capillaries/mm^2^), Ab-HepIII (359±61 capillaries/mm^2^), or Ab-RGD (373±70 capillaries/mm^2^) had statistically significantly higher capillary density compared either to the PBS treatment group (196±54 capillaries/mm^2^) or to Ab-FC/HV (255±36 capillaries/mm^2^). There was no statistical difference in capillary density among rats treated with Ab-HepI, Ab-HepIII, or Ab-RGD. Rats treated with Ab only (185±34 arterioles/mm^2^) did not induce any differences in capillary formation compared to PBS treatment group. Both the Ab only and the PBS treatment groups served as negative controls.

Arteriogenesis (Figure 3b) in rats treated with Ab-HepI (10±2 arterioles/mm^2^), Ab-HepIII (9±1 arterioles/mm^2^), or Ab-RGD (10±2 arterioles/mm^2^) showed statistically significantly higher arteriole density when compared to the PBS treatment group (5±2 arterioles/mm^2^) or to Ab-FC/HV (7±1). There also was no statistical difference in the arteriole density among rats treated with Ab-HepI, Ab-HepIII, or Ab-RGD. We observed no difference in arteriole density between PBS-treated rats and rats treated with Ab only (5±1 arterioles/mm^2^).

**Figure 4:**
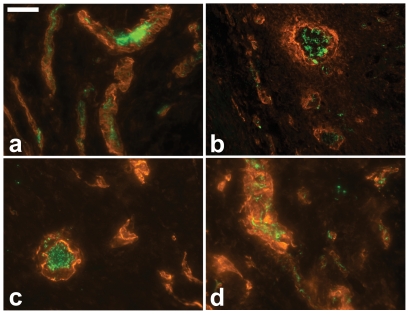
Microbead perfusion. High magnification (40×) images within the MI region showing the perfusion of 0.2 µm fluorescent microbeads (green) into arterioles that have been stained using anti-α smooth muscle actin (orange) in hearts treated with (**a**) Ab-FC/HV, (**b**) Ab-RGD, (**c**) Ab-HepI, or (**d**) Ab-HepIII. Scale bar: 15 µm.

To determine if the newly formed vessels were contiguous with the existing vasculature, we used previously established protocols whereby we perfused the hearts with fluorescently labeled microbeads [22], [23]. In Figure 4 we show overlaid high magnification images of regions within the myocardial infarct showing the fluorescent microbeads, indicated in green, within the lumen of the arterioles stained immunofluorescently using anti-α-smooth muscle actin. The presence of the microbeads indicated that the observed arterioles in the infarct region were functionally connected to pre-existing vessels.

Both Masson's trichrome- and hemotoxylin-and-eosin- (H&E) stained slides were used to assess infarct size. We did not find a statistically significant difference in the mean infarct size when compared to the PBS treatment group (Figure 3c).

### Affects of Targeted ECM peptides on LV function

The functional effects of treatment with Ab-peptide were investigated by echocardiography at baseline (1–2 days post MI and before treatment) and 6 weeks after treatment (Table 2). LV ejection fraction and wall thickness significantly decreased in the control groups, while LV dilation significantly increased in the control groups, all of which were indicators of worsening LV function. Only HepIII treatment prevented the negative LV remodeling affects following the MI.

**Table 2:** Echocardiography data showing the internal comparisons between measurements taken before injection (baseline) and 6 weeks after injection.

Measured Parameters	Treatment	Baseline	6 weeks post	P
***Ejection fraction, %***	Ab-HepI	38.6±2.81	34.1±2.83	0.0068
	Ab-HepIII	38.8±6.00	35.5±3.41	0.184
	Ab-RGD	41.2±4.16	33.8±4.57	0.000186
	Ab-FC/HV	39.4±3.47	32.5±1.61	0.000000629
	Ab only	40.8±1.73	31.0±5.53	0.0023
	PBS	39.6±2.91	31.6±4.00	0.000425
***LV diastolic volume, mL***	Ab-HepI	0.5±0.1	0.7±0.1	0.00008
	Ab-HepIII	0.5±0.1	0.7±0.1	0.004
	Ab-RGD	0.4±0.1	0.7±0.1	0.000001
	Ab-FC/HV	0.5±0.1	0.8±0.1	0.00006
	Ab only	0.5±0.04	0.8±0.1	0.00005
	PBS	0.5±0.1	0.7±0.1	0.002
***LV systolic volume, mL***	Ab-HepI	0.3±0.1	0.5±0.1	0.00007
	Ab-HepIII	0.3±0.1	0.5±0.1	0.002
	Ab-RGD	0.3±0.1	0.5±0.1	0.000001
	Ab-FC/HV	0.3±0.1	0.5±0.1	0.000003
	Ab only	0.3±0.0	0.6±0.1	0.00002
	PBS	0.3±0.1	0.5±0.1	0.003
***Infarct wall thickness, cm***	Ab-HepI	0.22±0.016	0.19±0.018	0.0083
	Ab-HepIII	0.21±0.014	0.17±0.023	0.00018
	Ab-RGD	0.21±0.014	0.17±0.013	0.021
	Ab-FC/HV	0.21±0.017	0.16±0.029	0.0044
	Ab only	0.22±0.024	0.14±0.015	0.000001
	PBS	0.21±0.013	0.15±0.016	0.000000056

## Discussion

Our results demonstrate that antibody targeting of ECM-derived peptides to ischemically injured myocardium can effectively help to produce an ECM favorable for angiogenesis. A critical barrier to tissue regeneration is the lack of an adequate vascular network. The creation of a vascular bed in the infracted myocardium should allow for greater cell engraftment and survival [24], [25]. Therefore, this strategy of specifically targeting ECM-derived peptides to the ischemic myocardium may provide a more favorable microenvironment for cell transplantation and myocardial regeneration. The use of the ECM-derived peptides alone, without growth factors or cells, was sufficient to promote an angiogenic response in infarcted rat hearts. The induction of new vessel formation suggests that targeting active components of the ECM can influence the microenvironment and allow the body to act as its own bioreactor to regenerate vital structures of the myocardium.

The MHC antibody specifically targets the ECM peptides to the MI region in the heart (Figure S2). Nuclear imaging studies using I^125^-radiolabeled MHC-Ab showed that the majority of the MHC-Ab was concentrated within the MI of the heart and was still detectable within the MI 1 week post-injection. Unconjugated I^125^-radiolabeled peptides injected into the rats 1 day post-MI were detectable within the heart 3 hours post-injection, but only at trace levels 24 hours post-injection. Additionally, biodistribution analysis showed that the unconjugated peptides were predominantly in other organs—e.g. liver, intestines—instead of the heart (Figure S3). Not only did the MHC-Ab concentrate the peptides within the MI, but it also allowed the peptides to remain within the MI for longer periods of time. Hence, in order to expect any benefit from peptide treatment after acute MI, it was necessary for us to target them with an antibody. However, even though most of the antibody was targeted to the infarct region of the heart, there were still trace levels in other organs, which could result in neoplastic angiogenesis within these organs. Future studies will need to be conducted to fully assess the effect of these trace levels in other organ systems in producing angiogenesis.

Our *in vitro* data found three peptides—HepI, HepIII, RGD—that exhibited similar properties, although to a lesser extent, as their source proteins, particularly in terms of promoting endothelial cell adhesion, proliferation and haptotactic migration. Nanogram amounts of either HepIII or RGD were sufficient to promote significant movement of endothelial cells. This is the same order of magnitude of peptides that we injected into our rats. Using fluorescently labeled peptides, we had determined that our conjugation resulted in crosslinking ∼3 moles of peptide per mole of antibody. The presence of the ECM-derived peptides could promote the migration of endothelial cells to the infarct site.

Cells interact with the ECM via receptors, including integrins. Yet, these receptors only interact with certain regions of an ECM protein. Our Western blot analysis showed activation of Erk1/2 by HepI, HepIII, and RGD. Activation of the Erk1/2 signal transduction pathway in ECs is critical for EC proliferation and angiogenesis [26], [27], [28], [29]. HepIII has been shown to interact with α_2_β_1_ and α_3_β_1_ integrins, thereby promoting cell adhesion to the peptide [11], [30]. There is some evidence that α_2_, α_3_, and β_1_ integrin subunits can also interact with HepI [11]. RGD has been shown to interact with the integrin α_v_β_3_ [31], [32], [33]. Endothelial cells migrate via α_v_β_3_ integrins. The interaction of the cells via these integrins can trigger a cascade of signal transduction pathways, some of which could be involved in initiating angiogenesis and/or arteriogenesis. For instance, α_v_β_3_ is involved in the signaling of fibroblast growth factor 2 (FGF2), which is involved in various signaling pathways, including the activation of Erk1/2, which in turn activates the signaling pathways for angiogenesis and/or arteriogenesis [20]. Integrin α_2_β_1_ has been shown to support VEGF-stimulated signal transduction [19], which also includes Erk1/2 activation. Integrin α_3_β_1_-mediated adhesion has been shown to activate focal adhesion kinase (FAK) as well as Erk in keratinocytes [34]. Even though HepI, HepIII, and RGD did not promote activation of Erk1/2 to the same extent, their induced angiogenic responses in terms of capillary and arteriole formation were similar. This suggests that in addition to Erk1/2 activation, the ability to induce cell migration and cell proliferation are characteristics that are important if not more so in the promotion of the observed arteriogenesis and angiogenesis.

Furthermore, the ECM peptides may be influencing new vessel formation by interacting with other ECM proteins. Interestingly, HepIII peptides can interact with one another to form a polymer-like matrix (Figure S4). It is possible that a comparable situation is occurring in rats treated with HepIII and may explain why HepIII was the only peptide that was able to prevent further negative remodeling as indicated by the echocardiography data. The peptides do not necessarily have to be interacting with each other. They may be able to interact with surrounding ECM proteins in a similar manner to form a matrix, thus altering the material properties of the LV and preventing the negative remodeling associated with a MI [35], [36]. Also, earlier studies have shown that HepI can interact with whole Col IV [12]. The low dissociation equilibrium constant K_d_ = 1.66 nM, indicates that the binding affinity of HepI for Col IV is remarkably high. HepI's ability to interact strongly with Col IV may in some way help to contribute to the formation of neovessels in HepI-treated rats, as Col IV is known to be a major factor in the induction of new vessel formation and in the stabilization of these new vessels [17].

Despite previous reports [13], [16] showing that FC/HV could promote cell adhesion and migration, we did not observe such behavior in our studies with FC/HV. Based on our results, the peptide might promote transient adhesion, as evidenced by our observation of spread cells 1 day after incubation on FC/HV-treated plates. Although FC/HV did induce Erk1/2 activation, we observed no statistically significant increase in angiogenesis or arteriogenesis in the FC/HV-treated rats. It is possible that FC/HV is inducing Erk1/2 activation in the cells already present within the MI region, but the inability of the peptide to promote significant endothelial cell migration and cell proliferation as compared to the other 3 peptides studied may limit its ability to induce any dramatic neovascular formation.

In conclusion, Ab-targeted ECM-derived peptides can be used to alter the myocardial microenvironment and promote the induction of angiogenesis in the injury site after a MI. The exact mechanisms by which the ECM peptides induced the observed *in vivo* angiogenic response, however, warrant further study. Furthermore, from our echocardiography data only Hep III prevented negative remodeling of the LV following a MI, indicating that neovascularization alone is insufficient to get full recovery of LV function. Perhaps by combining this ECM peptide therapy with cell therapy, we may be able to get full restoration of cardiac function and tissue. Nevertheless, our results present a new non-invasive strategy for regenerative therapies and a tool for investigating tissue repair and regeneration.

## Materials and Methods

### Peptides

The peptides were synthesized by Commonwealth Biotechnologies Inc. (Richmond, Virginia). Amino acid analysis was performed on the peptides to verify the amino acid sequence. The reagents used to crosslink the peptide to the antibody were purchased from Pierce (Rockford, IL).

### Initial cell attachment and proliferation assays

For the adhesion studies, 96-well Immulon 1B plates (Fisher, Pittsburg, PA) were used. The whole ECM protein from which the peptide was derived was used as a positive control. PBS-treated wells were used as a negative control. Since previous studies had shown that negative versions—e.g. different amino acid sequence but with the same hydropathy value, truncated, and/or scrambled— of the peptides used here abrogated or lessened their ability to promote cell adhesion and proliferation, we decided not to include scrambled or truncated versions of the peptides in this study [12], [15], [16], [37]. 50 µL of the peptide or protein in PBS at various concentrations was added to each well and allowed to incubate at 37°C overnight, after which the wells were blocked for 2 hours at 37°C with 2 mg/mL bovine serum albumin (BSA) solution and later washed twice with PBS before addition of human umbilical vein endothelial cells (HUVECs; Lonza, Basel, Switzerland). HUVECs were used because they were readily available, easily cultured, and frequently used as an *in vitro* model for testing angiogenic potential [38], [39]. Cell adhesion were assessed after 30 minutes of incubation at 37°C, 5.0% CO_2_ followed by treatment with a MTS (3-(4,5-dimethylthiazol-2-yl)-5-(3-carboxymethoxyphenyl)-2-(4-sulfophenyl)-2H-tetrazolium, inner salt) tetrazolium/formazan assay (Promega, Madison, WI).

For the proliferation assays, untreated Co-Star 96-well plates (Fisher, Pittsburg, PA) were used. The coating protocol was the same as above. Proliferation was measured using a MTS tetrazolium/formazan assay after 1, 2, and 3 days of incubation at 37°C, 5.0% CO_2_.

### Haptotactic cell migration

Haptotactic migration was performed in triplicate and was assessed via a modified Boyden chamber (8 µm pore size, Corning, Acton, MA) using previously established protocols [40]. In brief, the lower chamber first was blocked with 10% BSA for at least 30 minutes at 37°C followed by several washings with PBS. The underside part of the membrane on the upper chamber was coated with increasing concentrations (0.5–300 µg/mL) of either the peptide or ECM protein and allowed to incubate for up to 30 minutes at 37°C and then allowed to air dry at room temperature under aseptic conditions. Basal cell media (Lonza, Basel, Switzerland) supplemented with 0.5% BSA was added to the lower chamber. 100 µL of HUVECs in the same media was added to the upper chamber. After incubating at 37°C, 5.0% CO_2_ for 6 hours, the cells on the membrane of the upper chamber were fixed with 4% paraformaldehyde followed by removal of the cells on the upper side of the membrane with a Q-tip. Finally, the membrane was carefully removed from the chamber, dipped in a solution of a 1∶4000 dilution of Hoechst 33342 (Invitrogen, Carlsbad, CA) and placed on a glass slide. 5 random pictures were taken of each membrane at 10× magnification using fluorescence microscopy (Nikon Eclipse E800) to determine the area cell density.

### Western blot analysis

Untreated 35 mm CoStar dishes were coated with 100 µg/mL of peptide or their source protein,blocked with 2 mg/mL BSA followed by several PBS washes. HUVECs were cultured on these plates for 3 days. Cells cultured on dishes treated with just PBS were used as a control. To isolate the protein, the cells were scraped off the plates using a cell scraper and washed twice with cold PBS. The cells were incubated in 20–40 µL of NP40 lysis buffer (Fisher, Pittsburg, PA) supplemented with protease inhibitor (BD BaculoGold, BD Biosciences, San Diego, CA) for 30 minutes at room temperature, vortexing every 10 minutes. The cell membranes were spun the cells down and the supernatant extracted.

The protein (10 µg/well) was run through a 12% Tris-glycine SDS-PAGE gel (Invitrogen, Carlsbad, CA) at a constant voltage of 125 V and transferred to the transfer membrane (Hybond-P, Amersham Biosciences, Piscataway, NJ) at a constant current of 400 mA. The membrane was blocked with 10% milk protein (Bio-Rad, Hercules, CA) at room temperature, then incubated with the primary antibody (1∶2000 dilution for total Erk1/2, Chemicon, Temecula, CA; 1∶1000 dilution for phospho-Erk1/2, Chemicon; or 1∶400 dilution for β-tubulin, Santa Cruz Biotechnology, Santa Cruz, CA) overnight at 4°C. After incubating with the secondary antibody (1∶5000 dilution of IgG-HRP, Chemicon, for Erk1/2 and 1∶20,000 dilution for β-tubulin) for 1 hour at room temperature, the membrane was treated with ECL Plus (Amersham Biosciences). The film was developed using a Konica SRX-101A developer. To strip the proteins, the membrane was treated with stripping buffer for 30 minutes at room temperature.

### Antibody isolation and peptide conjugation

Anti-MHC was isolated from HB-276 hybridoma (ATCC, Masassas, VA). The hybridomas were injected into Balb/c mice for ascites production. The ascites was then run through a column packed with Protein A beads (Pierce, Rockford, IL). After dialyzing against sterile PBS, the purity of the antibody was verified via SDS-PAGE electrophoresis, using 3–8% Tris-Acetate gel (Invitrogen, Carlsbad, CA), and its concentration was determined using a Bio-Rad protein assay kit (Bio-Rad, Hercules, CA).

We conjugated the peptide to the targeting antibody using carbodiimide chemistry using the 1-ethyl-3-[3-dimethylaminopropyl]carbodiimide hydrochloride (EDC, Pierce, Rockford, IL) crosslinker. EDC together with sulfo-NHS (Pierce, Rockford, IL) were first incubated with 2 mg/mL peptide. After exchanging to sterile PBS via a desalting column, the reacted peptide was incubated with 1 mg of antibody. Solutions used for the reactions were sterile filtered and autoclaved prior to use. Endotoxicity of the Ab-peptide was determined using the limulus amebocyte lysate (LAL) assay (Lonza, Basel, Switzerland). *In vitro* cell attachment, cell proliferation, and haptotactic migration assays were also performed on the Ab-peptides.

### Acute myocardial infarction model

All surgical procedures were approved by the Committee for Animal Research at the University of California San Francisco. Female Sprague-Dawley rats (225–250 g) underwent occlusion of the left anterior descending coronary artery for 25 minutes before reperfusion as previously described [35], [41], [42]. The rats were randomized 1–2 days after MI to either control or treatment groups, and were given one intravenous injection via the external jugular vein. 500 µl (∼100 µg of total protein) of Ab-peptide, Ab only (negative control), or PBS (control) was injected into the rats. Each group consisted of 10 animals. Transthoracic echocardiography was performed 1–2 days post-MI and 6 weeks post-treatment as previously described [42], [43]. Following echocardiography at 6 weeks, the hearts were perfused with fluorescently labeled microbeads (Molecular Probes, Eugene, OR) according to previously described protocols [22], [23].

### Histology, immunohistochemistry, and immunofluorescence

Immediately after the microbead perfusion, the hearts were removed, rinsed in cold saline, blotted-dry and fresh frozen in Tissue Tek O.C.T. freezing medium (Sakura Finetek, Torrance, CA). The hearts were sectioned into 10 µm slices. Representative sequential slides were stained with Masson's trichrome stain and H&E for determination of infarct size as previously described [35]. Angiogenesis in the infarct was examined by immunohistochemical (IHC) staining with mouse monoclonal anti-CD31 (BD Biosciences Pharmingen, San Diego, CA) to visualize capillaries and with mouse monoclonal anti-α-smooth muscle actin (Sigma, St. Louis, MO) to detect arterioles [44]. The staining assay was performed using Mouse-on-rat HRP-polymer (Biocare Medical, Concord, CA) using slides that were sequential to the slides stained with trichrome and H&E. Capillaries in the infarct region were identified as a single layer of CD31-positive cells with flattened morphology. Vessel density was calculated on the basis of 5 high magnification fields per section that spanned the infarct and averaged among 5 sections for each sample. Arterioles within or bordering the infarct were identified as staining positive for α-smooth muscle actin and as having a visible lumen with a diameter between 10 and 100 µm [35], [45]. Arteriole density was calculated as the average number of arterioles in the total infarct area, out of 5 representative slides per sample.

### Statistical Analysis

Data are presented as mean ± standard deviation. Cell adhesion, proliferation, and migration measurements were compared using the student's *t* test. Differences between echocardiography measurements before and after injection were compared, using the paired *t* test. Differences in the echocardiography measurements, infarct size, and vessel counts across treatment groups were compared, using one-way analysis of variance ANOVA with Holm's adjustment. Significance was accepted as P<0.05.

## Supporting Information

Figure S1 (0.09 MB PDF)

Figure S2 (0.10 MB PDF)

Figure S3 (0.11 MB PDF)

Figure S4 (0.09 MB PDF)
