# Supplementary material for: Targeted In Vivo Extracellular Matrix Formation Promotes Neovascularization in a Rodent Model of Myocardial Infarction
Source: PLoS One. 2010 Apr 28;5(4):e10384. doi: 10.1371/journal.pone.0010384 (PMC2860995; doi:10.1371/journal.pone.0010384)

Figure S1. Cell morphology. To assess cell morphology, untreated 35 mm CoStar dishes were coated with with 100 µg/mL of **(a)** Col IV, **(b)** Hep I, **(c)** Hep III, **(d)** FN, **(e)** FC/HV, or **(f)** RGD. The whole ECM protein was used as the positive control, and PBS-treated wells were treated as a negative control. Each dish was seeded with ~20,000 HUVECs. Shown here are after 1 day of incubation at 37°C, 5.0% CO_2. The cells were observed with an inverted light microscope (Nikon Eclipse TE300).

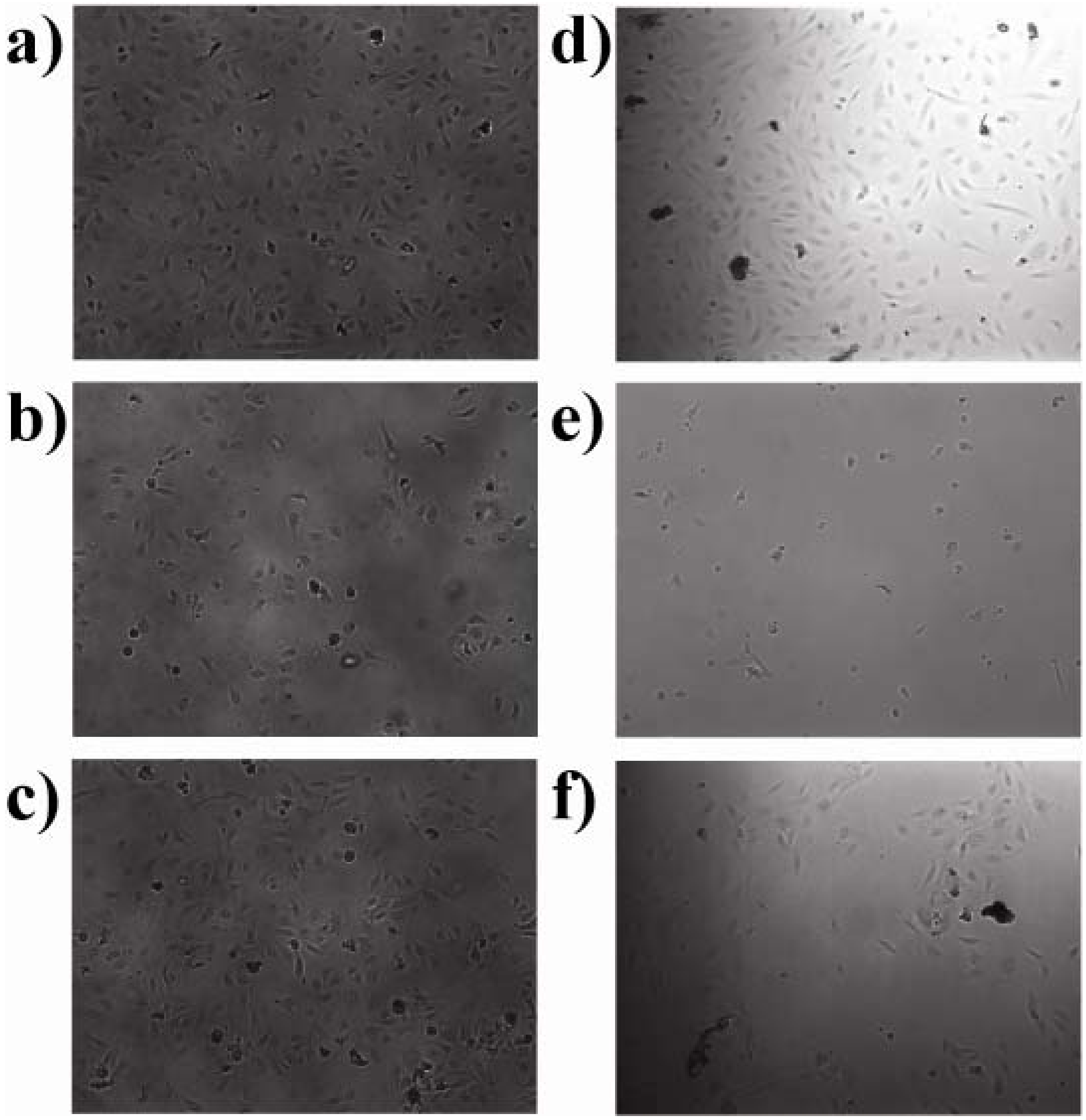

Supplement: Figure S1 — (0.09 MB PDF) [file pone.0010384.s001.pdf]
