# Supplementary material for: Targeted In Vivo Extracellular Matrix Formation Promotes Neovascularization in a Rodent Model of Myocardial Infarction
Source: PLoS One. 2010 Apr 28;5(4):e10384. doi: 10.1371/journal.pone.0010384 (PMC2860995; doi:10.1371/journal.pone.0010384)

Figure S2. Nuclear imaging studies of the Ab. 1 day post-MI rats were injected with 99, 216, or 231 µCi of ^{125}I labeled Ab via the jugular vein. The rats were then sacrificed 1 day post-injection of the radiolabeled Ab, and the organs were excised, perfused with 1% w/v of 2,3,5-Triphenyltetrazolium chloride (TTC, Sigma) for 15 minutes at 37° C, and frozen. Biodistribution curves were established using digital autoradiograph analysis and tissue analysis. For tissue analysis, the whole organs were weighed and then counted via an Automatic Gamma Counter (Wallac Wizard 3'', PerkinElmer Life and Analytic Sciences, CT). Percentage of injected dose of radioactivity per gram (% ID/g) was determined using the following equations: % ID/g = 100 x Ti/A_s and A_s= I x B x D; where Ti is tissue activities; A_s is activities at specific time point; I is net injected dose; B is background for correction; D is tracer decay factor for calibration. For the autoradiographic analysis, the tissues were sliced (20 µm thickness) with a microtone. The slices were then exposed on the phosphor imager plate for 72 hours, and scanned with a Molecular Dynamics Phosphor Imager (Molecular Dynamics, Sunnyvale, CA). The regional radioactivities from the tissue sections were determined as an average activity per pixel. To determine infarct size, the heart slices with TTC staining were mounted on cardboard and scanned via a flatbed scanner. ImageJ was used to measure the area of infarction versus the area of left ventricle. TTC staining showed a sizeable infarct (white region) **(a)** and the autoradiograph showed the myosin heavy chain antibody is located within the infarct region **(b)**. Biodistribution analysis from the autoradiographs showed the antibody tended to be located predominantly within the region of infarct **(c)** and **(d)**.

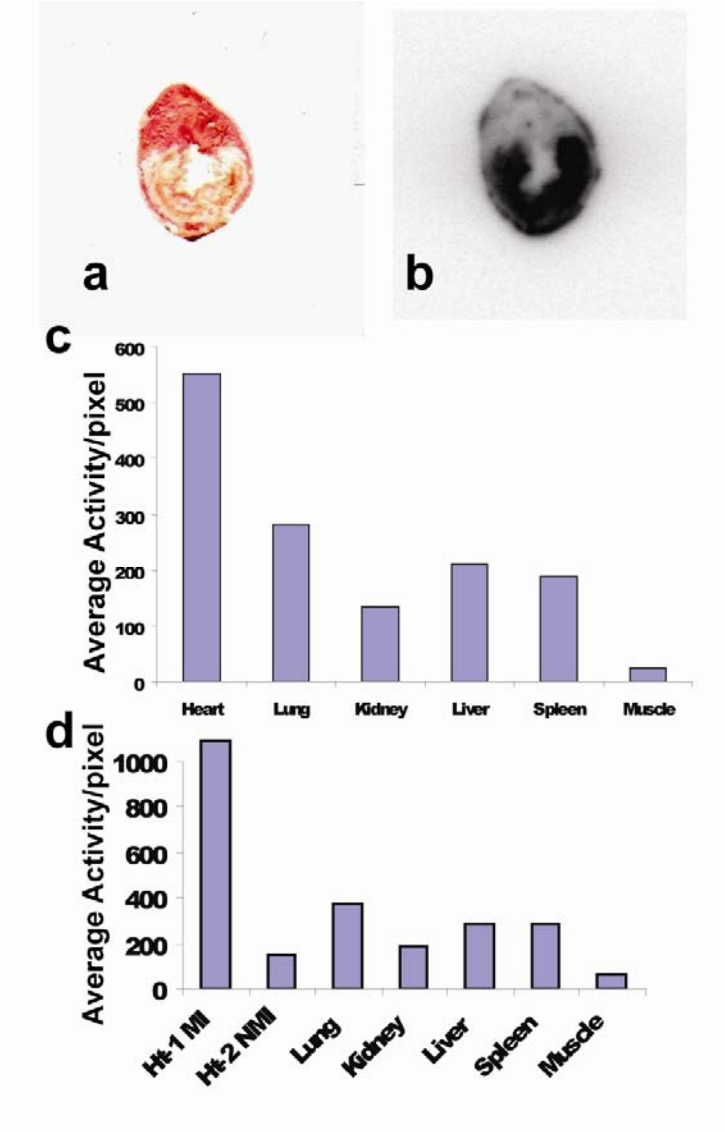

Supplement: Figure S2 — (0.10 MB PDF) [file pone.0010384.s002.pdf]
