# Supplementary material for: Targeted In Vivo Extracellular Matrix Formation Promotes Neovascularization in a Rodent Model of Myocardial Infarction
Source: PLoS One. 2010 Apr 28;5(4):e10384. doi: 10.1371/journal.pone.0010384 (PMC2860995; doi:10.1371/journal.pone.0010384)

Figure S3. Nuclear imaging studies of unconjugated HepI and HepIII. 1 day post-MI, rats were injected with 40 - 100 μCi of ^{125}I labeled HepI or HepIII via the jugular vein. The radiolabeling was performed via the lysine groups of the peptides. We were not able to radiolabel the RGD and FC/HV peptides since both lacked lysine groups. The rats were sacrificed either 3 hours or 24 hours post-injection of the radiolabeled peptides, and the organs were excised and frozen. Analysis on the organs was performed as described in Figure S2. **(a)** Biodistribution analysis from the autoradiographs of organs excised 3 hours post injections shows that HepI was mostly found within the blood and large intestine and HepIII within the liver, lung, and large intestine. **(b)** Biodistribution analysis from the autoradiographs of organs excised 24 hours post injections shows that HepI did not show any particular preference for any organ and HepIII was mostly found within the liver, lung, and spleen.

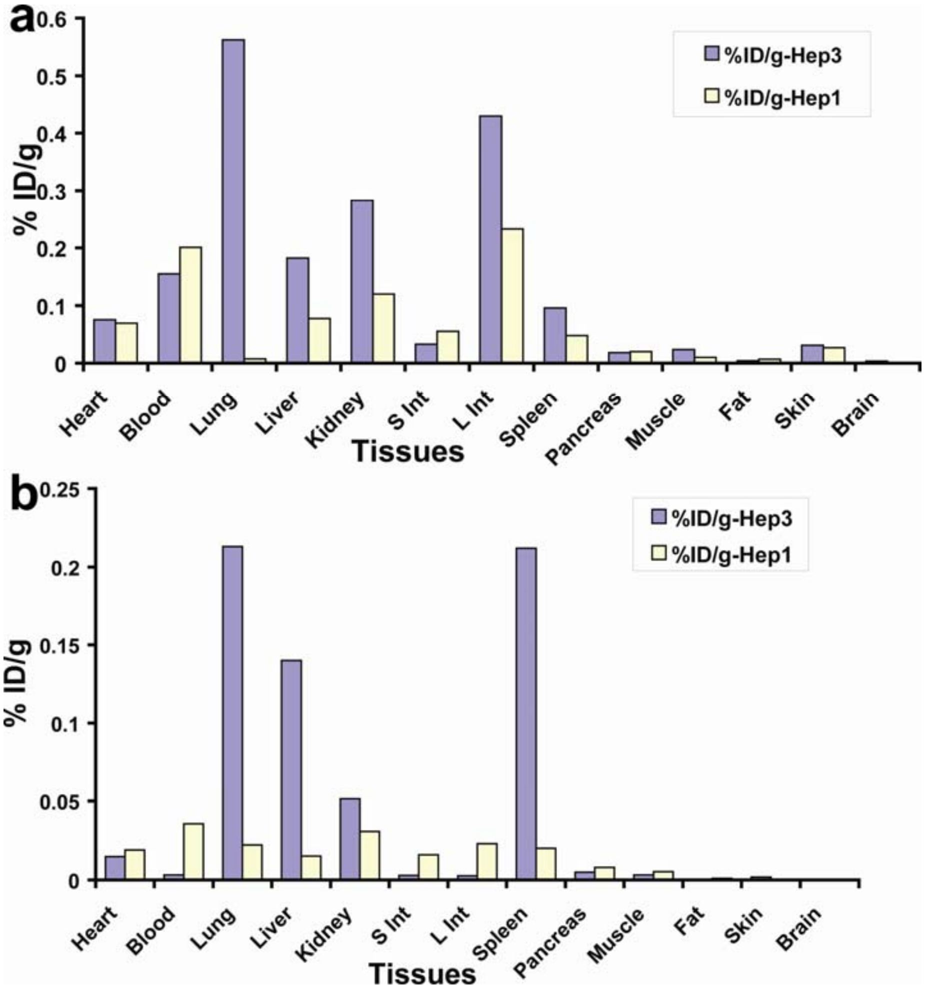

Supplement: Figure S3 — (0.11 MB PDF) [file pone.0010384.s003.pdf]
