# Supplementary material for: Targeted In Vivo Extracellular Matrix Formation Promotes Neovascularization in a Rodent Model of Myocardial Infarction
Source: PLoS One. 2010 Apr 28;5(4):e10384. doi: 10.1371/journal.pone.0010384 (PMC2860995; doi:10.1371/journal.pone.0010384)

**Figure S4.** Transmission electron microscope (TEM) image of Hep III. A drop of saturated peptide solution was added to a grid that had been coated with Formvar (polyvinyl formal). Fixation, staining, and imaging of the grids with the electron microscope were done by the Research Morphology Core Facility (Department of Pathology and Laboratory Medicine, University of California, San Francisco). At sufficiently high enough concentrations, we were able to observe formation of a matrix.

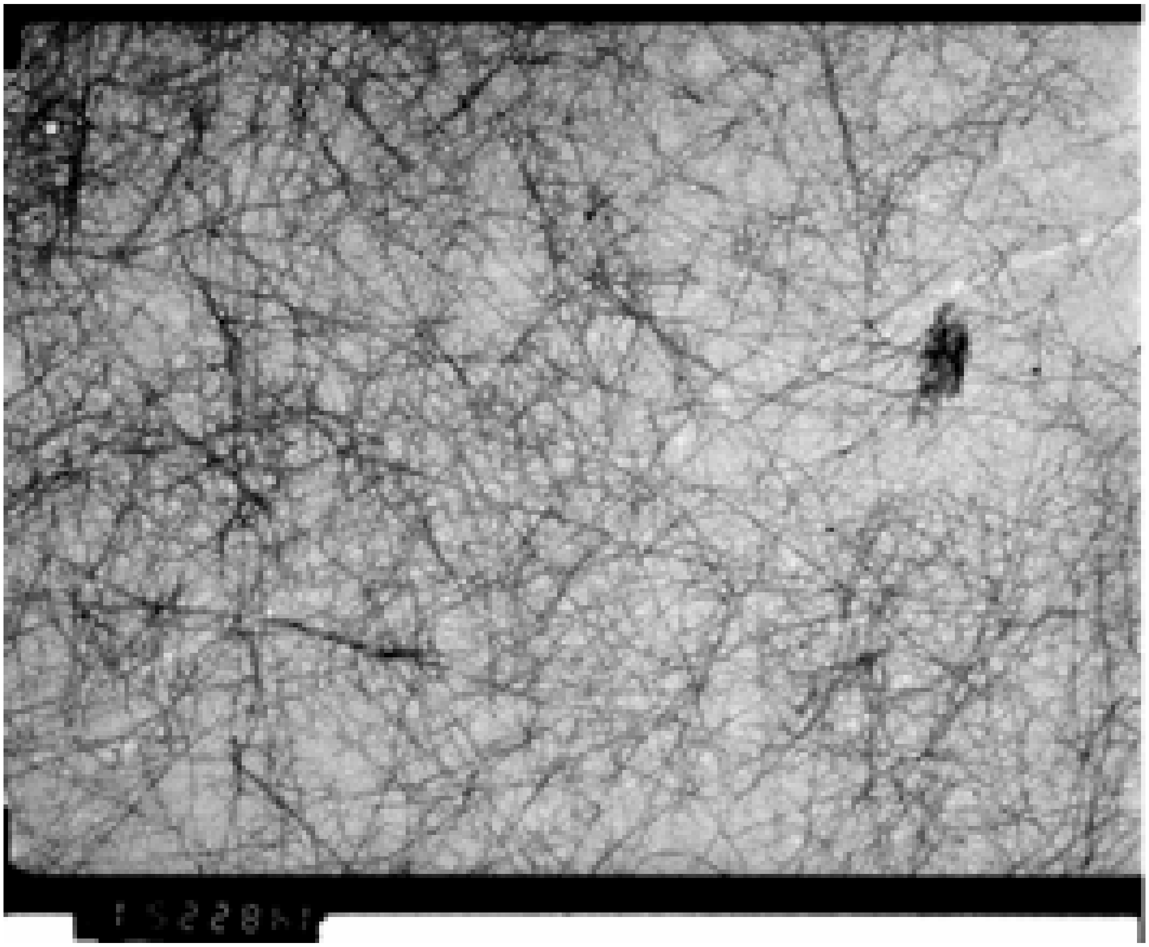

Supplement: Figure S4 — (0.09 MB PDF) [file pone.0010384.s004.pdf]
